# Practical Departments

**Published:** 1905-12-16

**Authors:** 


### PRACTICAL DEPARTMENTS.

### AN APPARATUS FOR AUTOMATICALLY UNLOCKING ALL DOORS FROM ANY PART OF THE BUILDING.

MESSRS. JOSEPH KAYE AND SONS, of Leeds, have brought out an apparatus which enables the responsible officer of an asylum to unlock all doors in the building from any convenient point. The arrangement can be fitted, we understand, to existing locks. It consists of a brass plate, forming the front plate of the lock, held in place by hooks on the ends of two levers. The hook levers move on pivots. When it is desired to open the doors, a bolt in the neighbourhood of the hook levers moves them so as to release the front plate, and the bolts of the lock can then be moved outwards by a slight pressure from inside, as in opening a door in the ordinary way. The bolt which moves the hook levers can be actuated by compressed air, water, or electricity, but has only been adopted for electricity up to the present. With the latter an electro-magnet is fixed in the neighbourhood of each lock and actuates the bolt when the circuit of its coils is closed, all the coils of all the electro-magnets being connected with a battery, and with ordinary bell pushes at every point from which it is desired to control the opening of the doors. If compressed air or, as it is usually termed, the pneumatic system is employed, the bolt is actuated by a piston in a small cylinder, which is connected with pushes fixed at every point from which control is to be obtained, the pushes being in the form of the usual pneumatic bell compressor, an air vessel, arranged to be compressed when the push button is forced in. An apparatus worked by electricity has been in use at Wakefield Asylum for some time, and is stated to be giving every satisfaction. A model was shown at the recent Electrical Exhibition at Olympia.

